# Edaravone Dexborneol Alleviates Cerebral Ischemic Injury via MKP-1-Mediated Inhibition of MAPKs and Activation of Nrf2

**DOI:** 10.1155/2022/4013707

**Published:** 2022-09-06

**Authors:** Wen Zhang, Haiguang Yang, Mei Gao, Hengai Zhang, Lili Shi, Xiaoyan Yu, Rui Zhao, Junke Song, Guanhua Du

**Affiliations:** ^1^State Key Laboratory of Bioactive Substances and Functions of Natural Medicines, Institute of Materia Medica, Chinese Academy of Medical Sciences and Peking Union Medical College, Beijing 100050, China; ^2^Beijing Key Laboratory of Drug Target Identification and Drug Screening, Institute of Materia Medica, Chinese Academy of Medical Sciences and Peking Union Medical College, Beijing 100050, China

## Abstract

The edaravone and dexborneol concentrated solution for injection (edaravone-dexborneol) is a medication used clinically to treat neurological impairment induced by ischemic stroke. This study was aimed at investigating the preventive effects and the underlying mechanisms of edaravone-dexborneol on cerebral ischemic injury. A rat four-vessel occlusion (4-VO) model was established, and the neuronal injury and consequent neurological impairment of rats was investigated. Brain tissue malondialdehyde (MDA), myeloperoxidase (MPO), and nitric oxide (NO) levels were determined. The levels of proteins in mitogen-activated protein kinases (MAPKs), nuclear factor erythroid 2-related factor 2 (Nrf2), and nuclear factor-*κ*B (NF-*κ*B) signaling pathways were determined by western immunoblotting. The function of mitogen-activated protein kinase phosphatase 1 (MKP-1) was investigated using both western blot and immunofluorescence methods, and the effect of the MKP-1 inhibitor, (2*E*)-2-benzylidene-3-(cyclohexylamino)-3*H*-inden-1-one (BCI), was investigated. The results indicated that edaravone-dexborneol alleviated neurological deficiency symptoms and decreased apoptosis and neuron damage in the hippocampal CA1 area of the ischemic rats. Edaravone-dexborneol increased the MKP-1 level; decreased the phosphorylation of extracellular signal-regulated kinase (ERK), c-Jun N-terminal kinase (JNK), and p38 mitogen-activated protein kinase (p38 MAPK); inhibited NF-*κ*B p65 activation; and boosted Nrf2 activation, all of which were partially reversed by the MKP-1 inhibitor, BCI. The above results indicated that the upregulation of MKP-1 contributed to the protective effects of edaravone-dexborneol against ischemic brain injury. Our findings support the hypothesis that edaravone-dexborneol can alleviate cerebral ischemic injury via the upregulation of MKP-1, which inhibits MAPKs and activates Nrf2.

## 1. Introduction

Stroke is one of the leading causes of death and disability in humans, and caring for stroke patients has imposed a tremendous burden on society [1]. The most common type of stroke is ischemic stroke with complex clinical manifestations. Currently, there is a shortage of drugs for the prevention and treatment of ischemic stroke. Although the thrombolytic agent tissue plasminogen activator (t-PA) is successful in treating cerebral thrombosis, only a small number of patients can receive it in time due to its limited therapeutic window [2]. Thus, developing novel drugs for the treatment of ischemic stroke is of great urgency.

The edaravone and dexborneol concentrated solution for injection (edaravone-dexborneol (Eda-Bor)) is a compound preparation approved in China for the treatment of acute ischemic stroke. It is formulated from edaravone and (+)-borneol in a mass ratio of 4 : 1. Edaravone is a radical scavenger with the effects of quenching hydroxyl radicals and clearing lipid peroxidation [3, 4]. Edaravone has been approved and suggested for the treatment of acute ischemic stroke in China and Japan [5, 6]. In addition, edaravone was recently approved by the United States Food and Drug Administration (FDA) for the treatment of amyotrophic lateral sclerosis (ALS), which slowed the disease progression of ALS clinically [7]. A recent meta-analysis of clinical trials indicated that edaravone ameliorated neurological impairment with a survival benefit at three-month follow-up, regardless of the mean age or duration of treatment [8]. Borneol itself has been demonstrated to have neuroprotective benefits [9], especially in cerebral ischemic rats by improving nerve function and decreasing cerebral infarction [10]. Additionally, borneol is classed as an adjuvant drug that facilitates targeting to the cerebral lesion sites. Borneol promoted the movement of drugs across the blood-brain barrier (BBB) and enhanced drug distribution into brain tissues, implying a synergistic effect in the treatment of acute ischemic stroke [11].

The combination of edaravone and borneol (Eda-Bor) with an optimal ratio of 4 : 1 has been clinically reported to be synergistic [12]. Eda-Bor was safe and well tolerated at dosages of 12.5, 37.5, and 62.5 mg in a phase II investigation compared to edaravone alone [13]. In the follow-up phase III comparative trial, a total of 1165 acute ischemic stroke patients were randomly assigned to the Eda-Bor group (*n* = 585) or the edaravone group (*n* = 580). On day 90 after randomization, the Eda-Bor group (37.5 mg) had a significantly greater proportion of patients with favorable functional outcomes than the edaravone group [14]. The combination of edaravone and borneol has been demonstrated to be beneficial in clinical trials. In addition to the known ROS scavenging effects, an in-depth elucidation of its mechanism of action and the involved signaling pathways still needs to be done to guide clinical practice. The purpose of this study was to confirm the efficacy and explore the mechanism of Eda-Bor action in a rat four-vessel occlusion (4-VO) model.

## 2. Material and Methods

### 2.1. Animals and Drugs

Male Sprague Dawley (SD) rats (240-280 g) were purchased from Beijing Vital River Laboratory Animal Technology Co., Ltd. with the certificate number SCXK (Beijing) 2006-0009. The rats were kept at a temperature of 23-25°C with free access to food and water.

Eda-Bor injection (batch number: S090904-02-121, 181-210108, 12.5 mg/5 mL) and edaravone injection (batch number: 80-090703, 10 mg/5 mL) were both provided by Jiangsu Simcere Pharmaceutical Research Co., Ltd. Each bottle of Eda-Bor injection (12.5 mg/5 mL) contains 10 mg edaravone and 2.5 mg dexborneol with a mass ratio of 4 : 1. (2*E*)-2-Benzylidene-3-(cyclohexylamino)-3*H*-inden-1-one (BCI), a mitogen-activated protein kinase phosphatase 1 (MKP-1) inhibitor [15], was obtained from the Institute of Materia Medica, Chinese Academy of Medical Sciences and Peking Union Medical College (CAMS & PUMC).

### 2.2. Animal Grouping, 4-VO Model Establishment, and Drug Administration

Rats were divided into seven groups, including the sham operation group, 4-VO model group, 4-VO+Eda-Bor (0.375, 0.75, and 1.5 mg/kg) groups, 4-VO+edaravone (3 mg/kg) group, and 4-VO+Eda-Bor (1.5 mg/kg)+BCI group. All the animal procedures were reviewed and approved by the Institutional Animal Care and Use Committee of the Institute of Materia Medica, CAMS & PUMC.

The rat 4-VO model was established according to the reported procedure with some modifications [16]. The 4-VO model has been commonly used to explore the mechanism of brain damage following transient global ischemia but can also be used for testing neuroprotective drugs. To establish the 4-VO model, two vertebral arteries of rats were permanently coagulated, and two common carotid arteries were temporarily ligated. The 4-VO model induces transient ischemia to the forebrain, while maintaining a relatively intact blood supply to the hindbrain. In this study, after the rat was anesthetized and fixed, the bilateral vertebral arteries were occluded by electrocauterization. After 24 h, the rats were anesthetized again, and the bilateral common carotid arteries were clamped with clips to initiate the ischemic period. If the ischemic operation was successful, the rats experienced loss of consciousness, corneal reflex and righting reflex, dilated pupils, and gray or white eyeballs; the consciousness and the above reflexes did not recover after the ischemic period. After 20 min, the clips were removed to start the reperfusion period. Rats that died during the ischemic period and those that had convulsions throughout the experiment were discarded. The sham operation group did not have bilateral vertebral artery coagulation or bilateral common carotid artery clamping, while the remaining operations were the same. Except for the sham operation group and model group, the rats were only given vehicle. The other groups were given the indicated drugs once a day for 3 days after reperfusion. BCI was administered intraperitoneally once a day at 0, 24, and 48 h after reperfusion initiation. Each dose of BCI was 2.5 mg/kg in saline containing 5% dimethyl sulfoxide (DMSO) and 1.25% Tween 20 [17].

### 2.3. Modified Neurological Severity Scores

At 24 and 72 h after the start of reperfusion, a researcher who was blind to the experimental protocol assessed the rats' neurological function. The scoring system was as previously described and included balance, sensory, motor, and reflex tests [18], and the score ranged from 0 to 18 (normal, 0; highest deficit, 18). An increase in the score indicated a more serious injury. Each time a rat failed to perform the test or failed to display a tested reflex, one point was scored.

### 2.4. Pathological Examination of Neurons in the Hippocampal CA1 Area

Rats were anesthetized at 72 h after reperfusion and were perfused and fixed with 4% paraformaldehyde. The brains were removed, placed in paraformaldehyde, and fixed for ≥3 days followed by routine dehydration and paraffin embedding. 5 *μ*m thick brain sections were cut and stained with cresyl violet. A light microscope was used to photograph the CA1 region of the hippocampus in both hemispheres. The histological alterations in the hippocampal subfield were rated using the grading method described in the previous paper [19]. The following criteria were applied: 0 indicates normal neuron morphology with no damage; 1 indicates a few neurons are injured; 2 indicates a large number of neurons are damaged; and 3 indicates the majority of neurons are damaged. Neuronal density was measured by counting neurons with intact cell membranes, full nucleus, and clear nucleolus in the hippocampal CA1 area on both sides under high magnification (×400). Additionally, the average number of undamaged neurons per unit length in the CA1 region of the hippocampus was determined.

### 2.5. Determination of MDA, MPO, and NO Content in the Brain Tissue

Kits for quantitation of malondialdehyde (MDA), myeloperoxidase (MPO), and nitric oxide (NO) were purchased from Nanjing Jiancheng Bioengineering Institute, China. At 24 h after reperfusion, the entire brain tissue of some of the rats was homogenized in 2 mL of ice-cold buffer containing 50 mM Tris-HCl, 150 mM NaCl, 5 mM CaCl_2_, and 0.1 mM phenylmethylsulfonyl fluoride (PMSF) at pH 7.4. Samples were centrifuged at 12,000 g for 20 min at 4°C. The supernatants were recovered, and MDA, MPO, and NO concentrations were determined following the kit instructions.

### 2.6. Western Blotting

After 72 h of reperfusion, western blotting of brain tissue protein was performed. Sodium dodecyl sulfate-polyacrylamide gel electrophoresis (SDS-PAGE) was used to separate brain proteins, which were subsequently transferred to a polyvinylidene difluoride (PVDF) membrane. After blocking the membrane with 5% bovine serum albumin (BSA), the proteins were incubated with the following primary antibodies: MKP-1, cleaved caspase-3, Bax, Bcl-2, p38 mitogen-activated protein kinase (p38 MAPK), p-p38 MAPK, c-Jun N-terminal kinase (JNK), p-JNK, extracellular signal-regulated protein kinase (ERK), p-ERK1/2, nuclear factor erythroid 2-related factor 2 (Nrf2), heme oxygenase-1 (HO-1), NAD(P)H:quinone oxidoreductase 1 (NQO1), nuclear factor-*κ*B (NF-*κ*B) p65, p-NF-*κ*B p65, NF-*κ*B inhibitor alpha (I*κ*B*α*), p-I*κ*B*α*, *β*-actin, and Histone H3 at 4°C overnight. The PVDF membrane was then washed with tris-buffered saline plus Tween 20 (TBST) and incubated for 2 h at 37°C with horseradish peroxidase- (HRP-) conjugated secondary antibody (anti-rabbit, 1 : 1000; anti-mouse, 1 : 5000). An enhanced chemiluminescence (ECL) kit was used to detect protein bands.

### 2.7. Immunofluorescence Assay

Immunofluorescence experiments were performed 72 h after reperfusion. For MKP-1 staining, the brain tissue was fixed in a 4% paraformaldehyde. Afterwards, frozen slices of 5 *μ*m thickness were made. Brain slices were then treated with 0.5% Triton X-100 and 10% goat serum, after which they were incubated overnight with primary antibodies against MKP-1 and NeuN. After washing, the sections were incubated with fluor-tagged secondary antibodies, and fluorescent images of MKP-1 and NeuN staining were captured under a microscope following DAPI treatment.

### 2.8. Statistical Analysis

The data are presented as the mean ± standard deviation (SD). To determine the differences between the study groups, a one-way analysis of variance (ANOVA) followed by Tukey's post hoc test was utilized. Statistical significance was defined as *P* < 0.05.

## 3. Results

### 3.1. Edaravone-Dexborneol Alleviated Neurological Deficits in 4-VO Rats

**Figure 1:**
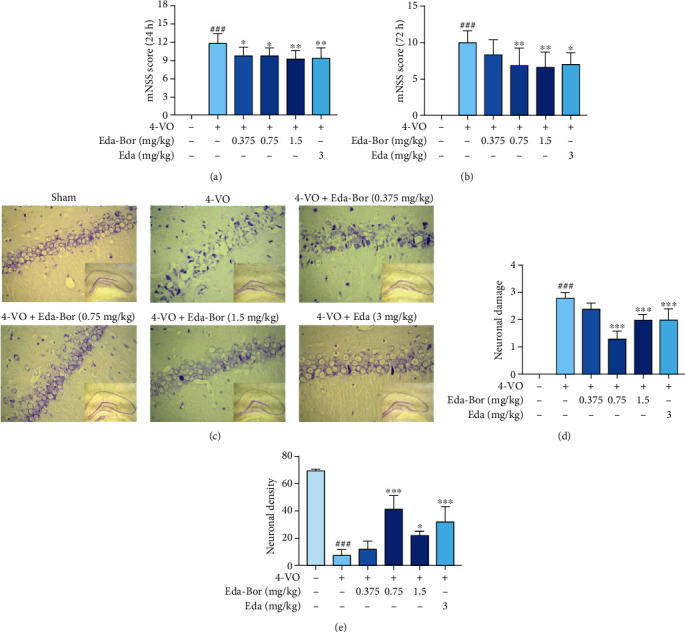
Edaravone-dexborneol alleviated neurological impairments and decreased pathological damage in the hippocampal CA1 region of rats. (a) At 24 h after reperfusion, Eda-Bor improved the neurological impairments of rats. (b) At 72 h after reperfusion, Eda-Bor improved the neurological impairments of rats. (c) Eda-Bor significantly reduced the pathological damage in the CA1 region, as determined by cresyl violet staining. (d) Eda-Bor reduced neuronal injury in the CA1 region. (e) Eda-Bor increased the neuronal density in the CA1 region. For the neurological deficits test, sham operation group: *n* = 10, 4-VO model group: *n* = 9, 4-VO+Eda-Bor (0.375 mg/kg) group: *n* = 9, 4-VO+Eda-Bor (0.75 and 1.5 mg/kg) groups: *n* = 8, and 4-VO+edaravone group (3 mg/kg): *n* = 8. For cresyl violet staining, sham operation group: *n* = 4, 4-VO model group: *n* = 5, 4-VO+Eda-Bor (0.375 mg/kg) group: *n* = 6, 4-VO+Eda-Bor (0.75 mg/kg) groups: *n* = 7, 4-VO+Eda-Bor (1.5 mg/kg) group: *n* = 6, and 4-VO+edaravone group (3 mg/kg): *n* = 7. Data were expressed as the mean ± SD. ^###^*P* < 0.001 compared with the sham operation group, ^∗^*P* < 0.05,  ^∗∗^*P* < 0.01, and^∗∗∗^*P* < 0.001 compared with the 4-VO model group. Eda-Bor: edaravone-dexborneol; Eda: edaravone.

The model group demonstrated significant neurological deficits at 24 and 72 h compared to the sham operation group (*P* < 0.001, Figures 1(a) and 1(b)). At 24 h following reperfusion, the Eda-Bor groups (0.375, 0.75, and 1.5 mg/kg) and the edaravone group showed significant improvement in neurological deficiency symptoms: *P* < 0.05, *P* < 0.05, *P* < 0.01, and *P* < 0.001, respectively (Figure 1(a)). At 72 h after reperfusion, the Eda-Bor injection groups (0.75 and 1.5 mg/kg) and the edaravone group showed beneficial effects on neurological deficiency symptoms: *P* < 0.01, *P* < 0.01, and *P* < 0.05 (Figure 1(b)). The findings revealed that cerebral ischemia damage resulted in significant neurological impairments, which were significantly alleviated by the administration of Eda-Bor. Additionally, the effect of Eda-Bor in improving neurological deficiency symptoms was better than the positive control, edaravone, used alone.

### 3.2. Edaravone-Dexborneol Reduced Injury to the Hippocampal CA1 Region in 4-VO Rats

Brain slices were stained with cresyl violet 72 h after 4-VO surgery. The CA1 region of the hippocampus was pathologically graded, and the neurons were counted using a light microscope (×400). As shown in Figures 1(c) and 1(d), cresyl violet staining revealed no obvious alteration in the hippocampal CA1 region of the sham-operation group; the neuronal damage grade was 0. The neurons were neatly aligned with intact morphology, including large, spherical nuclei, and distinct nucleoli. However, most neurons in the CA1 region of 4-VO rats were damaged or disrupted, and the remainder had ischemic alterations with elongated shapes and pyknotic or absent nuclei, with a pathogenic grade of 2.8 ± 0.2 (Figure 1(d)). Eda-Bor (0.75 and 1.5 mg/kg) significantly decreased the pathological damage induced by the 4-VO operation (*P* < 0.001 and *P* < 0.001) (Figure 1(d)) and increased neuronal density (*P* < 0.001 and *P* < 0.05) (Figure 1(e)). The positive control, 3 mg/kg edaravone, also reduced the 4-VO-induced damage.

### 3.3. Edaravone-Dexborneol Decreased the MDA, MPO, and NO Levels in the Brain Tissue of 4-VO Rats

**Figure 2:**
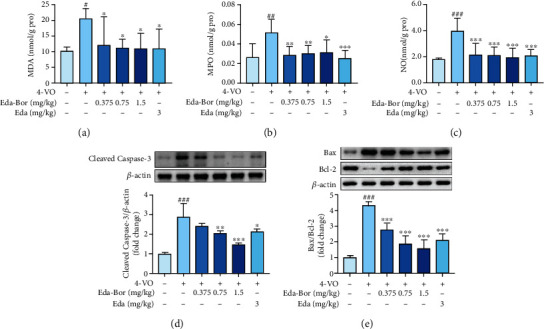
Edaravone-dexborneol decreased the levels of MDA (a), MPO (b), NO (c), cleaved caspase-3 (d), and Bax/Bcl-2 ratio (e) in the brain tissue of 4-VO rats. For the determination of MDA, MPO, and NO, sham operation group: *n* = 5, 4-VO model group: *n* = 8, 4-VO+Eda-Bor (0.375 and 0.75 mg/kg) groups: *n* = 8, 4-VO+Eda-Bor (1.5 mg/kg) groups: *n* = 7, and 4-VO+edaravone group (3 mg/kg): *n* = 8. For the western blot method, *n* = 4. Data were expressed as the mean ± SD. ^#^*P* < 0.05,  ^##^*P* < 0.01, and^###^*P* < 0.001 compared with the sham operation group. ^∗^*P* < 0.05,  ^∗∗^*P* < 0.01, and^∗∗∗^*P* < 0.001 compared with the 4-VO model group. Eda-Bor: edaravone-dexborneol; Eda: edaravone.

MDA, MPO, and NO levels in brain tissue of 4-VO rats were elevated 24 h after reperfusion: *P* < 0.05, *P* < 0.01, and *P* < 0.001 (Figures 2(a)–2(c)). Eda-Bor (0.375, 0.75, and 1.5 mg/kg) decreased the levels of MDA in brain tissue dose-dependently (*P* < 0.05) (Figure 2(a)). The administration of Eda-Bor at 0.375, 0.75, and 1.5 mg/kg resulted in a decrease in MPO levels: *P* < 0.01, *P* < 0.01, and *P* < 0.05 (Figure 2(b)). The concentration of NO was also decreased dose-dependently by Eda-Bor at 0.375, 0.75, and 1.5 mg/kg (*P* < 0.001) (Figure 2(c)). As a positive control, edaravone inhibited the increase of MDA, MPO, and NO: *P* < 0.05, *P* < 0.001, and *P* < 0.001 (Figures 2(a)–2(c)).

### 3.4. Edaravone-Dexborneol Inhibited Apoptosis in the Brain Tissue of 4-VO Rats

As illustrated in Figures 2(d) and 2(e), the intensity of cleaved caspase-3 and Bax was increased in the injured brain region, whereas the intensity of Bcl-2 was decreased. The Eda-Bor (0.75 and 1.5 mg/kg) and the edaravone groups significantly reduced cleaved caspase-3 intensity: *P* < 0.01, *P* < 0.001, and *P* < 0.05 (Figure 2(d)). Eda-Bor at 0.375, 0.75, and 1.5 mg/kg significantly reduced the Bax/Bcl-2 ratio (*P* < 0.001) (Figure 2(e)).

### 3.5. Edaravone-Dexborneol Suppressed MAPK Signaling and Activated Nrf2 Signaling in the Brain Tissue of 4-VO Rats

**Figure 3:**
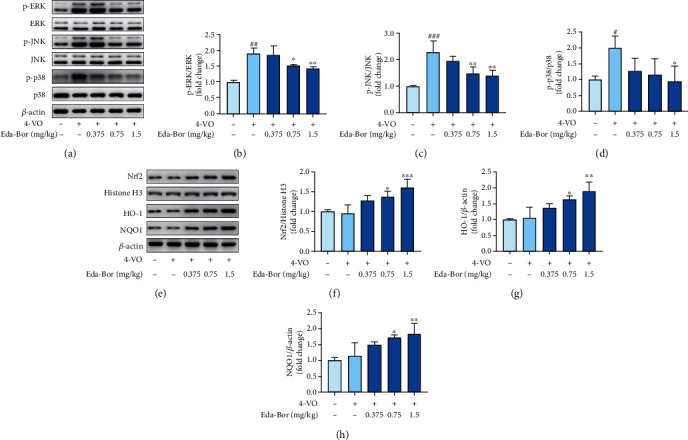
Edaravone-dexborneol inhibited MAPK signaling and activated Nrf2 signaling in brain tissue of 4-VO rats. (a) p-ERK, ERK, p-JNK, JNK, p-p38, and p38 levels. (b) Eda-Bor lowered the p-ERK/ERK ratio. (c) Eda-Bor the p-JNK/JNK ratio. (d) Eda-Bor lowered the p-p38/p38 ratio. (e) The Nrf2, NQO1, and HO-1 levels. (f) Eda-Bor increased the Nrf2 level. (g) Eda-Bor increased the expression of HO-1. (h) Eda-Bor increased the expression of NQO1. Western blot analysis was used to determine the protein levels adjusted to *β*-actin or Histone H3 (nuclear). Data were expressed as the mean ± SD. *n* = 4. ^#^*P* < 0.05 and^###^*P* < 0.001 compared with the sham operation group. ^∗^*P* < 0.05,  ^∗∗^*P* < 0.01, and^∗∗∗^*P* < 0.001 compared with the 4-VO model group. Eda-Bor: edaravone dexborneol; Eda: edaravone.

The mitogen-activated protein kinases (MAPKs) have been shown to play important roles in cerebral ischemia injury. The 4-VO injury increased the phosphorylation of ERK (*P* < 0.001) (Figures 3(a) and 3(b)), whereas Eda-Bor at 0.75 and 1.5 mg/kg significantly decreased the phosphorylation of ERK (*P* < 0.05 and *P* < 0.01) (Figures 3(a) and 3(b)). Enhanced phosphorylation of JNK was also observed following 4-VO injury, and Eda-Bor at 0.75 and 1.5 mg/kg significantly downregulated it (*P* < 0.01) (Figures 3(a) and 3(c)). The phosphorylation of p38 was increased in the model group (*P* < 0.05), but was significantly decreased by 1.5 mg/kg Eda-Bor (*P* < 0.05) (Figures 3(a) and 3(d)). Eda-Bor at 0.75 and 1.5 mg/kg significantly increased Nrf2 levels (*P* < 0.05 and *P* < 0.001) (Figures 3(e) and 3(f)). In addition, the Nrf2 downstream protein, HO-1, was upregulated by Eda-Bor at 0.75 and 1.5 mg/kg (*P* < 0.05 and *P* < 0.001) (Figures 3(e) and 3(g)). Treatment with Eda-Bor (0.75 and 1.5 mg/kg) also increased the expression of the downstream protein, NQO1 (*P* < 0.05 and *P* < 0.01) (Figures 3(e) and 3(h)).

### 3.6. Edaravone-Dexborneol Inhibited NF-*κ*B Activation in the Brain Tissue of 4-VO Rats

**Figure 4:**
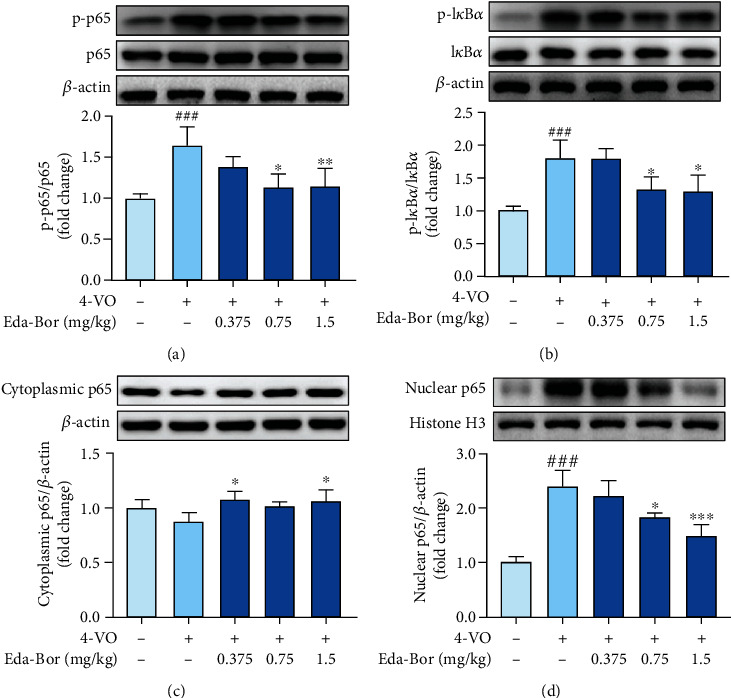
Edaravone-dexborneol inhibited the activation of NF-*κ*B in the brain tissue of 4-VO rats. (a) Eda-Bor lowered the p-p65/p65 ratio. (b) Eda-Bor lowered the p-I*κ*B*α*/I*κ*B*α* ratio. (c) Levels of p65 in the cytoplasm. (d) Eda-Bor decreased p65 levels in the nucleus. Data were expressed as the mean ± SD. *n* = 4. ^###^*P* < 0.001 compared with the sham operation group. ^∗^*P* < 0.05,  ^∗∗^*P* < 0.01, and^∗∗∗^*P* < 0.001 compared with the 4-VO model group. Eda-Bor: edaravone dexborneol; Eda: edaravone.

According to the western blotting results, NF-*κ*B p65 and I*κ*B*α* phosphorylation levels were dramatically enhanced (*P* < 0.001) (Figures 4(a) and 4(b)). Eda-Bor at 0.75 and 1.5 mg/kg significantly suppressed NF-*κ*B p65 phosphorylation (*P* < 0.01) (Figure 4(a)) and reduced I*κ*B*α* phosphorylation (*P* < 0.05) (Figures 4(b)).  Figure 4(c) showed that the cytoplasmic p65 level was slightly elevated in the Eda-Bor groups at 0.375 and 1.5 mg/kg compared to the 4-VO model group, indicating a reduction in p65 nuclear translocation. The nuclear translocation of p65 was significantly increased by 4-VO injury (*P* < 0.001), but was inhibited by 0.75 and 1.5 mg/kg Eda-Bor (*P* < 0.05 and *P* < 0.01) (Figure 4(d)).

### 3.7. Edaravone-Dexborneol Regulated MAPK, Nrf2, and NF-*κ*B Signaling through Upregulation of MKP-1 in the Brain Tissue of 4-VO Rats

**Figure 5:**
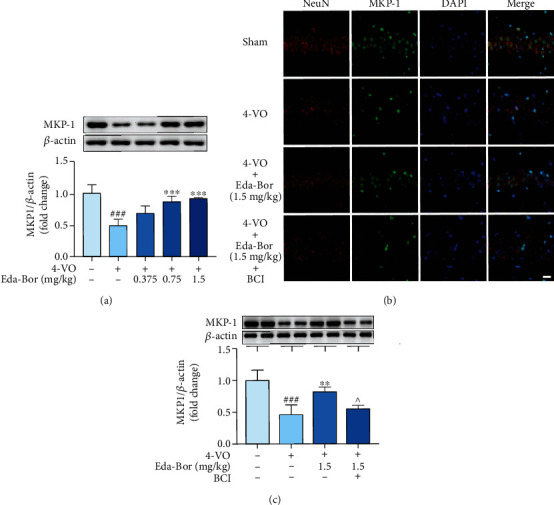
BCI partially reversed the MKP-1 upregulation effect of edaravone-dexborneol in brain tissue of 4-VO rats. (a) Eda-Bor dose-dependently inhibited 4-VO-induced downregulation of MKP-1. (b) Immunofluorescence analysis revealed that BCI partially reversed the increase in MKP-1 induced by Eda-Bor. (c) Western blot analysis revealed that BCI partially reversed the increase in MKP-1 induced by Eda-Bor. Data were expressed as the mean ± SD. *n* = 4. ^###^*P* < 0.001 compared with the sham operation group. ^∗∗^*P* < 0.01 and^∗∗∗^*P* < 0.001 compared the with 4-VO model group. ^^^*P* < 0.05 compared with the 4-VO+Eda-Bor (1.5 mg/kg) group. Eda-Bor: edaravone-dexborneol; Eda: edaravone. Scale bar = 20 *μ*m.

**Figure 6:**
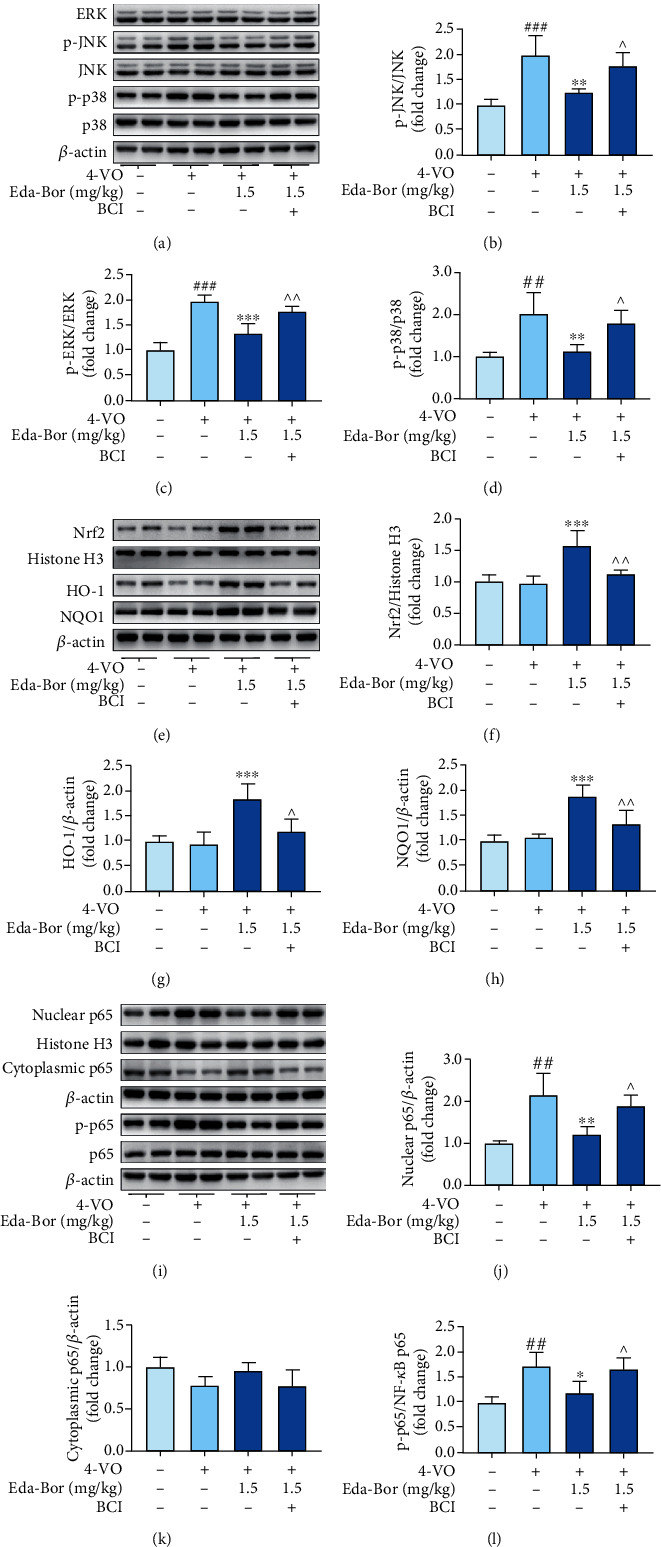
Edaravone-dexborneol injection suppressed the phosphorylation of MAPKs (a–d), promoted the activation of Nrf2 (e–h), and inhibited the activation of NF-*κ*B p65 (i–l) in the brain tissue, which were partially reversed by the MKP-1 inhibitor BCI. Data were expressed as the mean ± SD. *n* = 4. ^##^*P* < 0.001 and^###^*P* < 0.001 compared with the sham operation group. ^∗∗^*P* < 0.01 and^∗∗∗^*P* < 0.001 compared with the 4-VO model group. ^∧^*P* < 0.05 and^∧∧^*P* < 0.05 compared with the 4-VO+Eda-Bor (1.5 mg/kg) group. Eda-Bor: edaravone dexborneol; Eda: edaravone.

As determined by western blot and immunofluorescence, MKP-1 expression was dramatically decreased following 4-VO damage (Figures 5(a)–5(c)). Eda-Bor dose-dependently increased the MKP-1 level (Figure 5(a)). BCI, an MKP-1 inhibitor, was able to counteract the overexpression of MKP-1 induced by Eda-Bor (Figures 5(b) and 5(c)). Administration of 1.5 mg/kg Eda-Bor increased MKP-1 expression in neurons (*P* < 0.01) (Figure 5(b)), which was reversed by BCI (*P* < 0.05) (Figure 5(b)). Additionally, Eda-Bor suppressed the phosphorylation of MAPKs (Figures 6(a)–6(d)), enhanced Nrf2 activation (Figures 6(e)–6(h)), and inhibited NF-*κ*B p65 activation (Figures 6(i)–6(l)), all of which were partially reversed by the MKP-1 inhibitor, BCI.

## 4. Discussion

In this study, Eda-Bor alleviated the symptoms of neurological impairments in 4-VO rats and inhibited apoptosis and neuron damage in the hippocampal CA1 region of rats. The combination of edaravone and dexborneol is superior to the use of edaravone alone. Eda-Bor suppressed the phosphorylation of MAPKs, inhibited the activation of NF-*κ*B p65, and promoted the activation of Nrf2. These regulatory effects were mediated through the upregulation of MKP-1 by Eda-Bor.

Eda-Bor is a compound medicine containing edaravone and borneol in a mass ratio of 4 : 1. Edaravone exhibited high radical-scavenging activity against a broad spectrum of radical species, including the hydroxyl radical, superoxide anion, singlet oxygen, methyl radical, alkoxyl radical, and alkylperoxyl radical [20, 21]. Since cerebral ischemic injury induces the generation of many different types of radical species, the broad scavenging activity of edaravone is important for its therapeutic effects on ischemic stroke. In addition to its effects on radical elimination, edaravone suppressed both cerebral and systemic inflammatory responses [22, 23], decreased matrix metalloproteinase levels [24], and inhibited cell apoptosis [25]. Borneol usually acts as an adjuvant that facilitates drugs across various physiological barriers, such as the BBB [26]. The concentration of edaravone in the brain tissue was increased by pretreatment with borneol [27]. Although borneol is rarely used alone in the therapy of brain illnesses, it does show neuroprotective effects when used by itself [28]. Borneol alone can reduce the damage caused by cerebral ischemia and alleviate the BBB disruption [29]. A previous study in a rat cerebral ischemic model reported that borneol (1 mg/kg) reduced brain infarct size, decreased neurological deficit scores, and dose-dependently inhibited the production of proinflammatory molecules such as inducible nitric oxide synthase (iNOS) and tumor necrosis factor alpha (TNF-*α*) [30]. Eda-Bor has significant antioxidative stress, anti-inflammatory response, and antiapoptotic effects. In our study, Eda-Bor lowered the levels of MDA, MPO, and NO in the brain tissue of 4-VO rats. Eda-Bor also decreased the levels of proapoptotic proteins Bax and cleaved caspase-3 and raised the level of antiapoptotic protein Bcl-2.

The combination of edaravone and dexborneol exhibited synergistic benefits. In the rat transient cerebral ischemia/reperfusion model, edaravone, borneol, or Eda-Bor lowered the infarct volume with maximum effects of 55.7%, 65.8%, and 74.3%, respectively [12]. Y-2 is a mixture of edaravone and (+)-borneol in a 5 : 1 weight ratio. In a rat model of intracerebral hemorrhage induced by collagenase IV injection, Y-2 (1, 3, and 6 mg/kg) improved sensorimotor dysfunction, reduced cell death, alleviated histological changes, decreased brain edema, and preserved the blood-brain barrier integrity. Y-2 was superior to edaravone in terms of efficacy [31]. Eda-Bor also inhibited interleukin-6 (IL-6) and cyclooxygenase-2 generation in RAW264.7 cells stimulated by LPS. In a mouse model of acute lung injury (ALI), Eda-Bor significantly lowered TNF-*α* and IL-6 levels in serum and bronchoalveolar lavage fluid and inhibited NF-*κ*B activation. In this ALI model, Eda-Bor was found to be more effective than edaravone alone [32]. In a mouse model of dextran sulfate sodium-induced colitis, Eda-Bor at 7.5 and 15 mg/kg exhibited better therapeutic effects in relieving the disease activity index, reducing the body weight loss, and decreasing the levels of inflammatory cytokines than edaravone or (+)-borneol alone [33].

MAPKs are a group of highly conserved serine/threonine protein kinases. ERK, JNK, and p38 MAPK are the three major subfamilies of MAPKs. The activation of MAPKs triggers important physiological processes such as inflammation, oxidative stress, and apoptosis [34]. Deactivating MAPKs can reduce apoptosis and the inflammatory response, thereby alleviating cerebral ischemia damage. A study reported that JNK was significantly activated after cerebral ischemia/reperfusion injury in aged rats, while edaravone (3 mg/kg, i.v.) treatment significantly inhibited oxidative stress and the JNK signaling pathway [35]. Another study revealed that edaravone protected the myocardium from cerebral ischemia/reperfusion-induced injury in elderly rats primarily by inhibiting p38 MAPK activation [36]. In HT22 cells, H_2_O_2_ increased the levels of p-ERK, p-JNK, and p-p38 significantly, while edaravone protected cells from H_2_O_2_-induced damage by reducing ROS generation and inhibiting MAPK activation [37]. Besides edaravone, borneol (100 mg/kg) alone significantly reduced brain neuronal and microglial inflammation in LPS-induced sepsis in mice by inhibiting p-p65 and p38 MAPK signaling [38]. In this study, Eda-Bor reduced cerebral ischemia-induced phosphorylation of ERK, JNK, and p38, thereby inhibiting MAPK-mediated apoptosis and inflammation and reducing brain damage.

MKP-1 is a member of the threonine-tyrosine dual-specificity phosphatase family. It is a key phosphatase responsible for the dephosphorylation/deactivation of the MAPKs. MKP-1 inhibited MAPK-mediated proinflammatory signaling pathways, thereby inhibiting oxidative stress and cell death in a variety of illnesses [39]. A study showed that overexpression of MKP-1 inhibited neuronal mortality *in vitro*, possibly by modulating JNK signaling [40]. MKP-1 protected neurons from damage both *in vivo* and *in vitro*, and upregulation of MKP-1 alleviated LPS-induced neuroinflammation [41]. The ability of MKP-1 to suppress p38, JNK, and ERK activation is key to its inhibition of inflammation and apoptosis [42]. In our study, the administration of Eda-Bor increased the MKP-1 level in the 4-VO rat model, suggesting a possible mechanism for Eda-Bor to alleviate ischemic injury.

MKP-1 could be deactivated by ROS, because after being oxidized, MKP-1 is rapidly degraded in the proteasome [43]. Recent studies showed that TGF-1 treatment of NIH3T3 cells for 1 h increased nuclear and cytosolic ROS generation, resulting in thiol alteration of the MKP-1 protein and a 50% reduction in MKP-1 activity [44]. Additionally, ROS-induced inactivation of MKP-1 may result in the prolonged activation of JNK and p38. Increased ROS production and decreased MKP-1 activity are responsible for the MAPK-triggered inflammation following cerebral ischemia. The scavenging of ROS by Eda-Bor could inhibit the degradation of MKP-1, which preserved its inhibitory effects on MAPKs.

MKP-1 could also enhance the activation of Nrf2. Nrf2 is a transcription factor involved in the expression of numerous cytoprotective proteins, such as HO-1 and NQO1. Once activated, Nrf2 translocates into the nucleus, resulting in the transcription of downstream protective genes. It was reported that elevated MKP-1 expression promoted Nrf2 nuclear translocation, which further elevated the mRNA expression levels of antioxidant enzymes [45]. MKP-1 could inhibit the inflammatory response by interfering with the Nrf2 signaling pathway [46]. A recent study showed that MKP-1 enhanced the stability of Nrf2 and positively regulated Nrf2/HO-1 expression by directly interacting with the DIDLID motif of Nrf2 [47]. The interaction between MKP-1 and Nrf2 enhanced the antioxidant ability of cells and protected against cerebral ischemia damage. Several studies have reported that edaravone promoted the nuclear translocation of Nrf2 in acute cerebral ischemia injury [48], chronic cerebral hypoperfusion injury [49], and retinal ischemia/reperfusion injury [50]. In A*β*-treated SH-SY5Y cells, borneol was also reported to increase the nuclear translocation of Nrf2 and promote the expression of HO-1 [51]. Our study revealed that Eda-Bor promoted Nrf2 activation, which was inhibited by the MKP-1 inhibitor BCI. The above findings suggested that MKP-1 might also be an essential protein for the activation of the Nrf2 signaling pathway by Eda-Bor.

The regulation of Eda-Bor on MKP-1 and its downstream signaling pathways were partially reversed by the MKP-1 inhibitor BCI. The reason that BCI only acted partially might be due to the relatively weak inhibitory effect of BCI on MKP-1, which might not be strong enough to be fully effective. A previous study reported that the half-maximal inhibitory concentration (IC_50_) of BCI on MKP-1 was 11.5 ± 2.8 *μ*M in cells [52]. Another study reported that BCI specifically inhibited MKP-1 with a half maximal effective concentration (EC_50_) of 8.0 ± 0.6 *μ*M in cells [15]. A more potent inhibitor might be able to exert full regulation. A second possible explanation for BCI's partial activity might be that the bioavailability and blood-brain barrier permeability of BCI could influence its regulatory effects on MKP-1 and the related downstream proteins. Third, although MKP-1 is a key protein for Eda-Bor to alleviate cerebral ischemic injury, other mechanisms might also contribute to the regulatory effects of Eda-Bor on the related signaling pathways. Further research needs to be done to investigate the above three possibilities.

## 5. Conclusion

**Figure 7:**
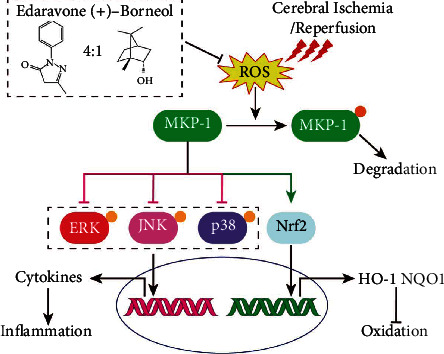
The mechanism of edaravone-dexborneol action in the treatment of cerebral ischemic injury.

The present work indicated that the combination of edaravone and dexborneol could protect 4-VO rats against ischemic brain injury. Eda-Bor inhibited rat neurological impairments by increasing the level of MKP-1. The upregulation of MKP-1 by Eda-Bor subsequently inhibited the activation of MAPKs and promoted the activation of Nrf2 (Figure 7). Upregulating MKP-1 activity offers a promising strategy for stroke treatment.

## Data Availability

The data applied and analyzed in the present study are available from the corresponding authors on reasonable request.

## References

[1] Pandian J. D., Gall S. L., Kate M. P. (2018). Prevention of stroke: a global perspective. *Lancet*.

[2] Chapman S. N., Mehndiratta P., Johansen M. C., McMurry T. L., Johnston K. C., Southerland A. M. (2014). Current perspectives on the use of intravenous recombinant tissue plasminogen activator (tPA) for treatment of acute ischemic stroke. *Vascular Health and Risk Management*.

[3] Edaravone Acute Infarction Study Group (2003). Effect of a novel free radical scavenger, edaravone (MCI-186), on acute brain infarction. Randomized, placebo-controlled, double-blind study at multicenters. *Cerebrovascular Diseases*.

[4] Watanabe K., Tanaka M., Yuki S., Hirai M., Yamamoto Y. (2018). How is edaravone effective against acute ischemic stroke and amyotrophic lateral sclerosis?. *Journal of Clinical Biochemistry and Nutrition*.

[5] Wang Y., Liu M., Pu C. (2017). 2014 Chinese guidelines for secondary prevention of ischemic stroke and transient ischemic attack. *International Journal of Stroke*.

[6] Shinohara Y., Yanagihara T., Abe K. (2011). II. Cerebral infarction/transient ischemic attack (TIA). *Journal of Stroke and Cerebrovascular Diseases*.

[7] Rothstein J. D. (2017). Edaravone: a new drug approved for ALS. *Cell*.

[8] Chen C., Li M., Lin L., Chen S., Chen Y., Hong L. (2021). Clinical effects and safety of edaravone in treatment of acute ischaemic stroke: a meta-analysis of randomized controlled trials. *Journal of Clinical Pharmacy and Therapeutics*.

[9] Liu R., Zhang L., Lan X. (2011). Protection by borneol on cortical neurons against oxygen-glucose deprivation/reperfusion: involvement of anti-oxidation and anti-inflammation through nuclear transcription factor *κ*appaB signaling pathway. *Neuroscience*.

[10] Xie Q., Li J., Dong T. (2022). Neuroprotective effects of synthetic borneol and natural borneol based on the neurovascular unit against cerebral ischaemic injury. *The Journal of Pharmacy and Pharmacology*.

[11] Zhu Q. Y., Tang S., Yang X. Q. (2022). Borneol enhances the protective effect against cerebral ischemia/reperfusion injury by promoting the access of astragaloside IV and the components of Panax notoginseng saponins into the brain. *Phytomedicine*.

[12] Wu H. Y., Tang Y., Gao L. Y. (2014). The synergetic effect of edaravone and borneol in the rat model of ischemic stroke. *European Journal of Pharmacology*.

[13] Xu J., Wang Y., Wang A. (2019). Safety and efficacy of Edaravone Dexborneol versus edaravone for patients with acute ischaemic stroke: a phase II, multicentre, randomised, double-blind, multiple-dose, active-controlled clinical trial. *Stroke and Vascular Neurology*.

[14] Xu J., Wang A., Meng X. (2021). Edaravone dexborneol versus edaravone alone for the treatment of acute ischemic stroke: a phase III, randomized, double-blind, comparative trial. *Stroke*.

[15] Korotchenko V. N., Saydmohammed M., Vollmer L. L. (2014). *In vivo* structure-activity relationship studies support allosteric targeting of a dual specificity phosphatase. *Chembiochem*.

[16] Pulsinelli W. A., Brierley J. B. (1979). A new model of bilateral hemispheric ischemia in the unanesthetized rat. *Stroke*.

[17] Liu L., Doran S., Xu Y. (2014). Inhibition of mitogen-activated protein kinase phosphatase-1 (MKP-1) increases experimental stroke injury. *Experimental Neurology*.

[18] Chen J., Li Y., Wang L. (2001). Therapeutic benefit of intravenous administration of bone marrow stromal cells after cerebral ischemia in rats. *Stroke*.

[19] Kato H., Liu Y., Araki T., Kogure K. (1991). Temporal profile of the effects of pretreatment with brief cerebral ischemia on the neuronal damage following secondary ischemic insult in the gerbil: cumulative damage and protective effects. *Brain Research*.

[20] Kamogawa E., Sueishi Y. (2014). A multiple free-radical scavenging (MULTIS) study on the antioxidant capacity of a neuroprotective drug, edaravone as compared with uric acid, glutathione, and trolox. *Bioorganic & Medicinal Chemistry Letters*.

[21] Amekura S., Shiozawa K., Kiryu C., Yamamoto Y., Fujisawa A. (2022). Edaravone, a scavenger for multiple reactive oxygen species, reacts with singlet oxygen to yield 2-oxo-3-(phenylhydrazono)-butanoic acid. *Journal of Clinical Biochemistry and Nutrition*.

[22] Zhang M., Teng C. H., Wu F. F. (2019). Edaravone attenuates traumatic brain injury through anti-inflammatory and anti-oxidative modulation. *Experimental and Therapeutic Medicine*.

[23] Fujiwara N., Som A. T., Pham L. D. (2016). A free radical scavenger edaravone suppresses systemic inflammatory responses in a rat transient focal ischemia model. *Neuroscience Letters*.

[24] Miyamoto N., Pham L. D., Maki T., Liang A. C., Arai K. (2014). A radical scavenger edaravone inhibits matrix metalloproteinase-9 upregulation and blood-brain barrier breakdown in a mouse model of prolonged cerebral hypoperfusion. *Neuroscience Letters*.

[25] Ding Y., Zhu W., Kong W., Li T., Zou P., Chen H. (2021). Edaravone attenuates neuronal apoptosis in hippocampus of rat traumatic brain injury model via activation of BDNF/TrkB signaling pathway. *Archives of Medical Science*.

[26] Kulkarni M., Sawant N., Kolapkar A., Huprikar A., Desai N. (2021). Borneol: a promising monoterpenoid in enhancing drug delivery across various physiological barriers. *AAPS PharmSciTech*.

[27] Gao C., Li X., Li Y., Wang L., Xue M. (2010). Pharmacokinetic interaction between puerarin and edaravone, and effect of borneol on the brain distribution kinetics of puerarin in rats. *The Journal of Pharmacy and Pharmacology*.

[28] Li Y., Ren M., Wang J. (2021). Progress in borneol intervention for ischemic stroke: a systematic review. *Frontiers in Pharmacology*.

[29] Chen Z. X., Xu Q. Q., Shan C. S. (2019). Borneol for regulating the permeability of the blood-brain barrier in experimental ischemic stroke: preclinical evidence and possible mechanism. *Oxidative Medicine and Cellular Longevity*.

[30] Chang L., Yin C. Y., Wu H. Y. (2017). (+)-Borneol is neuroprotective against permanent cerebral ischemia in rats by suppressing production of proinflammatory cytokines. *Journal of Biomedical Research*.

[31] Hua Y., Zhou L., Yang W. (2021). Y-2 reduces oxidative stress and inflammation and improves neurological function of collagenase-induced intracerebral hemorrhage rats. *European Journal of Pharmacology*.

[32] Zhang Z., Luo Z., Bi A. (2017). Compound edaravone alleviates lipopolysaccharide (LPS)-induced acute lung injury in mice. *European Journal of Pharmacology*.

[33] Zhang X., Xu F., Liu L. (2017). (+)-Borneol improves the efficacy of edaravone against DSS-induced colitis by promoting M2 macrophages polarization via JAK2-STAT3 signaling pathway. *International Immunopharmacology*.

[34] Son Y., Cheong Y. K., Kim N. H., Chung H. T., Kang D. G., Pae H. O. (2011). Mitogen-activated rotein kinases and reactive oxygen species: how can ROS activate MAPK pathways?. *Journal Of Signal Transduction*.

[35] Wen J., Watanabe K., Ma M. (2006). Edaravone inhibits JNK-c-Jun pathway and restores anti-oxidative defense after ischemia-reperfusion injury in aged rats. *Biological & Pharmaceutical Bulletin*.

[36] Watanabe K., Ma M., Wen J., Kodama M., Aizawa Y. (2007). Effects of edaravone in heart of aged rats after cerebral ischemia-reperfusion injury. *Biological & Pharmaceutical Bulletin*.

[37] Zhao Z. Y., Luan P., Huang S. X. (2013). Edaravone protects HT22 neurons from H_2_O_2_-induced apoptosis by inhibiting the MAPK signaling pathway. *CNS Neuroscience & Therapeutics*.

[38] Wang L., Liang Q., Lin A. (2019). Borneol alleviates brain injury in sepsis mice by blocking neuronal effect of endotoxin. *Life Sciences*.

[39] Moosavi S. M., Prabhala P., Ammit A. J. (2017). Role and regulation of MKP-1 in airway inflammation. *Respiratory Research*.

[40] Koga S., Kojima S., Kishimoto T., Kuwabara S., Yamaguchi A. (2012). Over-expression of map kinase phosphatase-1 (MKP-1) suppresses neuronal death through regulating JNK signaling in hypoxia/re-oxygenation. *Brain Research*.

[41] Jiao F., Wang Y., Zhang W. (2020). AGK2 alleviates lipopolysaccharide induced neuroinflammation through regulation of mitogen-activated protein kinase phosphatase-1. *Journal of Neuroimmune Pharmacology*.

[42] Owens D. M., Keyse S. M. (2007). Differential regulation of MAP kinase signalling by dual-specificity protein phosphatases. *Oncogene*.

[43] Kamata H., Honda S., Maeda S., Chang L., Hirata H., Karin M. (2005). Reactive oxygen species promote TNF*α*-induced death and sustained JNK activation by inhibiting MAP kinase phosphatases. *Cell*.

[44] Liu R. M., Choi J., Wu J. H. (2010). Oxidative modification of nuclear mitogen-activated protein kinase phosphatase 1 is involved in transforming growth factor *β*1-induced expression of plasminogen activator inhibitor 1 in fibroblasts. *The Journal of Biological Chemistry*.

[45] Kouam A. F., Yuan F., Njayou F. N. (2017). Induction of Mkp-1 and nuclear translocation of Nrf2 by limonoids from Khaya grandifoliola C.DC protect L-02 hepatocytes against acetaminophen-induced hepatotoxicity. *Frontiers in Pharmacology*.

[46] Zheng Z., Chen Y., Huang J., Deng H., Tang X., Wang X. J. (2019). Mkp-1 is required for chemopreventive activity of butylated hydroxyanisole and resveratrol against colitis-associated colon tumorigenesis. *Food and Chemical Toxicology*.

[47] Li J., Wang H., Zheng Z. (2018). Mkp-1 cross-talks with Nrf2/Ho-1 pathway protecting against intestinal inflammation. *Free Radical Biology and Medicine*.

[48] Wang D., Peng X., Yang A., He Y., Dong L., Lu H. (2021). Edaravone promotes nerve function recovery after acute cerebral infarction in rats via targeting Keap1-Nrf2/ARE. *Panminerva Medica*.

[49] Zhang D., Xiao Y., Lv P. (2018). Edaravone attenuates oxidative stress induced by chronic cerebral hypoperfusion injury: role of ERK/Nrf2/HO-1 signaling pathway. *Neurological Research*.

[50] Xu Y. P., Han F., Tan J. (2017). Edaravone protects the retina against ischemia/reperfusion-induced oxidative injury through the PI3K/Akt/Nrf2 pathway. *Molecular Medicine Reports*.

[51] Hur J., Pak S. C., Koo B. S., Jeon S. (2013). Borneol alleviates oxidative stress via upregulation of Nrf2 and Bcl-2 in SH-SY5Y cells. *Pharmaceutical Biology*.

[52] Molina G., Vogt A., Bakan A. (2009). Zebrafish chemical screening reveals an inhibitor of Dusp6 that expands cardiac cell lineages. *Nature Chemical Biology*.

